# *Ruthenibacterium lactatiformans* isolated from a human blood culture: a first report

**DOI:** 10.1186/s12879-024-09606-4

**Published:** 2024-07-15

**Authors:** Shougen Sumiyoshi, Shigeto Hamaguchi, Keigo Kimura, Katsuyuki Negishi, Koshi Ninomiya, Manabu Sasaki, Satoshi Kutsuna

**Affiliations:** 1 Division of Infection Control and Prevention, Osaka University, 2-15 Yamadaoka, Suita-Shi, Osaka, 565-0871 Japan; 2 Division of Fostering Required Medical Human Resources, Center for Infectious Disease Education and Research (CiDER), Osaka University, Osaka, Japan; 3 Department of Transformative Analysis for Human Specimen, Graduate School of Medicine, Osaka University, Osaka, Japan; 4 Laboratory for Clinical Investigation, Osaka University Hospital, Osaka, Japan; 5 Department of Neurosurgery, Osaka University Graduate School of Medicine, Osaka, Japan

**Keywords:** *Ruthenibacterium lactatiformans*, Blood culture, Vertebral osteomyelitis, Bacteraemia, MRSA, Satellite phenomenon, Anaerobic bacteria

## Abstract

**Background:**

*Ruthenibacterium lactatiformans,* a Gram-stain-negative, rod-shaped, obligate anaerobic bacterium of the Oscillospiraceae family, has not been previously reported in human infections. This study reports the first case of bacteraemia and potential vertebral osteomyelitis caused by *Ruthenibacterium lactatiformans*.

**Case presentation:**

An 82-year-old man with a history of diabetes, chronic renal failure, and prior spinal surgery for spondylolisthesis and spinal stenosis presented with fever and lower back pain. Magnetic resonance imaging revealed multiple vertebral osteomyelitis lesions. Initial blood cultures identified methicillin-resistant *Staphylococcus aureus* (MRSA), which prompted vancomycin treatment. However, repeated blood cultures not only confirmed persistent MRSA, but also detected Gram-negative bacilli (GNB). Despite surgical removal of the spinal hardware and antimicrobial therapy, the patient’s osteomyelitis worsened, necessitating transfer for further management. Subsequent analysis using 16S rRNA gene sequencing identified the GNB as *Ruthenibacterium lactatiformans.*

**Conclusions:**

This is the first documented instance of human infection with *Ruthenibacterium lactatiformans*, signifying its pathogenic potential in vertebral osteomyelitis. The involvement of anaerobic bacteria and the possibility of polymicrobial infections complicate the diagnosis and treatment of vertebral osteomyelitis. This report underscores the need for caution when identifying the causative organism and selecting an appropriate treatment.

## Introduction

*Ruthenibacterium* is a Gram-stain-negative, rod-shaped, obligate anaerobic bacterium that belongs to the family Oscillospiraceae [1]. There have been no reported cases of human infections caused by these bacteria, and their clinical significance and characteristics remain unknown. Here, we report a case of bacteraemia caused by *Ruthenibacterium lactatiformans* in a patient with vertebral osteomyelitis, highlighting its clinical course and treatment response.

## Case presentation

An 82-year-old man with a history of diabetes mellitus and chronic renal failure was admitted to Osaka University Hospital with fever and lower back pain that started four days before admission. One year prior, he had undergone spinal surgery for posterior lumbar interbody fusion at L5/S1 for spondylolisthesis and posterior decompression from L2 to L4 for lumbar spinal stenosis. Postoperatively, the patient developed vertebral osteomyelitis caused by methicillin-resistant *Staphylococcus aureus* (MRSA). This complication necessitated removal of the L5/S1 cage, autologous bone grafting, and posterior fixation from the L3-L4-sacral-alar-iliac.

**Fig. 1:**
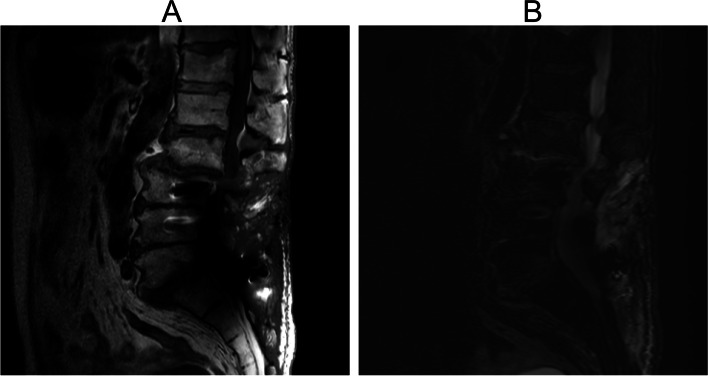
MRI findings of spine. **A** T1-weighted image, destruction of the vertebral endplates. **B** T2-weighted with fat suppression images, high signal intensity at the L2/3 level

Upon admission, magnetic resonance imaging (MRI) revealed fluid accumulation at the L2/3 and L5/S1 levels, with high signal intensity in the adjacent soft tissues (Fig. 1). Initial blood cultures identified MRSA on day 2 of admission, leading to vancomycin treatment. The time-to-positive (TTP) was 17 h.

Blood cultures were incubated for 7 days, and no additional bacteria were identified.

MRSA was detected in both aerobic and anaerobic bottles, and anaerobic culture was also performed, but no other bacteria were found. The patient was diagnosed as having vertebral osteomyelitis.

Blood samples revealed blood glucose level of 214 mg/dL and HbA1c of 6.2%.

**Fig. 2:**
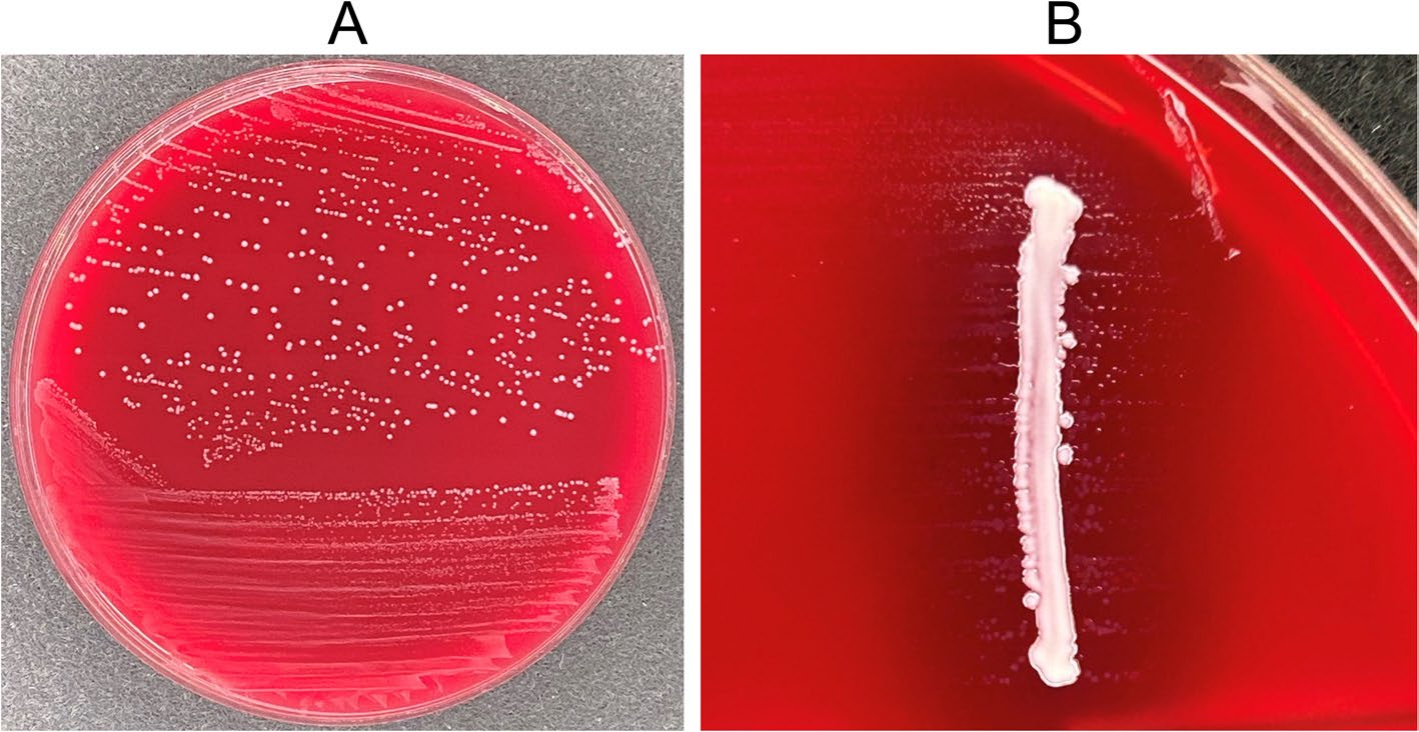
Culture colonies on blood agar plates. **A** On the plates of the blood culture-positive bottles, colonies of MRSA were seen, but few colonies of GNB. **B** The white line in the centre is a colony of MRSA, with smaller colonies scattered around it, a satellite phenomenon

On day 7 of admission, the patient underwent surgery to remove the spinal hardware. MRSA was also isolated from surgical specimens. Then, on day 10 of admission, MRSA continued to be detected in repeated blood cultures, with small Gram-negative bacilli (GNB) were identified in the two sets of blood cultures. The TTP of these cultures was 63 h. The colonies of GNB showed minimal growth (Fig. 2) and could not be identified by matrix-assisted laser desorption ionisation time-of-flight mass spectrometry (MALDI-TOF MS) using the MALDI Biotyper library (version 9) (Bruker Daltonics, https://www.bruker.com). We used ABHK media (Shimadzu Diagnostics) for the isolation of this microorganism. The isolation was performed under anaerobic conditions. To achieve anaerobiosis, we utilized the DIA anaero pack for pouches and DIA pouch bags (PHC). The cultures were incubated at 35 °C. Antimicrobial susceptibility testing for GNB was also unable because of the minimal growth. Under the combined treatment with vancomycin and ceftriaxone, the blood cultures turned negative on day 20 of admission. Since the bacteraemia improved, we determined to continue the ongoing therapy.

Ceftriaxone was administered for 16 days for GNB bacteraemia and vancomycin for eight weeks for the MRSA infection. Despite the completion of antibiotic treatment, the patient’s vertebral osteomyelitis worsened. Considering the possibility that GNB was the causative organism of vertebral osteomyelitis, ceftriaxone was resumed. On post-admission day 72, the patient was transferred for additional surgery. During the revision surgery, cultures from the vertebral body yielded only MRSA and no GNB were detected. Subsequent 16S rRNA gene sequence analysis identified the isolated GNB as *Ruthenibacterium lactatiformans* (GenBank accession number: JXXKH010000069) with 100% identity (1449/1449).

## Discussion

*Ruthenibacterium lactatiformans* was first identified in 2016 in the faeces of a healthy individual [1]. Despite several studies exploring its presence in the gut microbiota and faeces [2–4], no further infections have been reported to date. *Ruthenibacterium* belongs to the Oscillospiraceae family but exhibits Gram-negative staining. Disc-diffusion experiments have suggested that this organism is resistant to amikacin, ampicillin, azithromycin, cephalothin, clindamycin, levofloxacin, linezolid, and penicillin G, but sensitive to amoxicillin-clavulanate and vancomycin [1].

The causative organisms of vertebral osteomyelitis are reported to be anaerobic bacteria in less than 3% of cases [5, 6] and polymicrobial infections in 7.8% of cases [7]. Although infections involving polymicrobial bacteria are rare in vertebral osteomyelitis, they do occur. In particular, anaerobic bacteria are generally difficult to detect, which increases their risk of being overlooked.

In the case reported herein, vertebral osteomyelitis may have been caused by a coinfection involving *Ruthenibacterium lactatiformans* and MRSA. Therefore, great care must be taken during the pathogenesis and treatment of vertebral osteomyelitis.

Malignancy is a known risk factor for anaerobic bacteraemia [8, 9], and based on previous reports of *Ruthenibacterium lactatiformans* detected in human stool, the search for colorectal cancer as an entry point for this bacterium was considered. However, a colonoscopy could not be performed upon admission to our hospital because of the patient’s severe back pain.

## Conclusion

This case represents the first documented instance of rare bacteraemia and potentially vertebral osteomyelitis caused by *Ruthenibacterium lactatiformans*, providing significant insights into its pathogenic potential in human infections.

## Data Availability

The raw data of this article will be made available by the authors.

## Abbreviations

MRSA: Methicillin-resistant *Staphylococcus aureus*
MRI: Magnetic resonance imaging
GNB: Gram-negative bacilli
TTP: Time-to-positive
MALDI-TOF MS: Matrix-assisted laser desorption ionisation time-of-flight mass spectrometry
